# The association between the severity and distribution of white matter lesions and hemorrhagic transformation after ischemic stroke: A systematic review and meta-analysis

**DOI:** 10.3389/fnagi.2022.1053149

**Published:** 2022-11-25

**Authors:** Youjie Wang, Xueling Bai, Chen Ye, Yifan Yu, Bo Wu

**Affiliations:** ^1^West China School of Medicine, Sichuan University, Chengdu, China; ^2^Department of Neurology, West China Hospital, Sichuan University, Chengdu, China

**Keywords:** white matter lesion, leukoaraiosis, hemorrhage transformation, ischemic stroke, symptomatic intracerebral hemorrhage (sICH)

## Abstract

**Background and purpose:**

As a part of the natural course of ischemic stroke, hemorrhagic transformation (HT) is a serious complication after reperfusion treatment, which may affect the prognosis of patients with ischemic stroke. White matter lesions (WMLs) refer to focal lesions on neuroimaging and have been suggested to indicate a high risk of HT. This systematic review and meta-analysis aimed to summarize current evidence on the relation between WML and HT.

**Methods:**

This systematic review was prepared with reference to the Preferred Reporting Items for Systematic Reviews and Meta-Analyses (PRISMA) guidelines. We searched PubMed, Embase, Web of Science, and Cochrane Library databases for publications on WML and HT in patients with ischemic stroke. Odds ratios (ORs) and 95% confidence intervals (CIs) from eligible studies were combined to quantify the association between the severity of WML and the risk of HT. In addition, the descriptive analysis was adopted to evaluate the influence of different WML distributions on predicting HT.

**Results:**

A total of 2,303 articles were identified after removing duplicates through database searching, and 41 studies were included in our final analysis. The meta-analysis showed that the presence of WML was associated with HT (OR = 1.62, 95%CI 1.08–2.43, *p* = 0.019) and symptomatic intracerebral hemorrhage (sICH) (OR = 1.64, 95%CI 1.17–2.30, *p* = 0.004), and moderate-to-severe WML indicated a high risk of HT (OR = 2.03, 95%CI 1.33–3.12, *p* = 0.001) and sICH (OR = 1.92, 95%CI 1.31–2.81, *p* < 0.001). The dose–response meta-analysis revealed risk effects of increasing the severity of WML on both HT and ICH. In addition, both periventricular WML (PWML) (five of seven articles) and deep WML (DWML) (five of six articles) were shown to be associated with HT.

**Conclusions:**

White matter lesions are associated with overall HT and sICH in patients with ischemic stroke, and more severe WMLs indicate a high risk of HT and sICH. In addition, both PWML and DWMLs could be risk factors for HT.

**Systematic review registration:**

https://www.crd.york.ac.uk/prospero/, identifier: PROSPERO CRD42022313467.

## Introduction

Stroke is a major cause of long-term disability and death all over the world (Béjot et al., 2016). Currently, reperfusion therapies, including endovascular thrombectomy (EVT) and intravenous thrombolysis (IVT), are proven to be effective for patients with ischemic stroke as they can significantly improve clinical outcomes (Wardlaw et al., 2012; Emberson et al., 2014; Derex and Cho, 2017). However, as a part of the natural course of ischemic stroke, hemorrhagic transformation (HT) is also one of the mortal complications after reperfusion treatments (Hornig et al., 1986). Based on imaging features, HT ranges from smaller petechial hemorrhagic infarction (HI) to more confluent parenchymal hematoma (PH), and it can also be classified into symptomatic intracerebral hemorrhage (sICH) and asymptomatic hemorrhage according to the National Institute of Neurological Disorders and Stroke (NINDS), the Safe Implementation of Thrombolysis in Stroke-Monitoring Study (SITS-MOST), and the European Co-operative Acute Stroke Study-II (ECASS II) criteria (National Institute of Neurological Disorders Stroke rt-PA Stroke Study Group, 1995; Hacke et al., 1998; Wahlgren et al., 2008). Among these subtypes of HT, sICH is significantly associated with high mortality and poor outcomes among those patients (Yaghi et al., 2017). Thus, it is necessary to explore the association between some preexisting clinical features and the risk of HT to better treat patients with ischemic stroke.

According to previous studies, in addition to many clinical variables such as age, hypertension, and baseline stroke severity measured by the National Institute of Health Stroke Scale (NIHSS), neuroimaging markers of cerebral small-vessel disease (CSVD) were also found to be associated with HT (Emberson et al., 2014; Lei et al., 2022). As a common imaging biomarker of CSVD, white matter lesions (WMLs) are characterized by focal lesions without associated mass effect within the deep, subcortical, periventricular, or infratentorial white matter (Eikermann-Haerter and Huang, 2021), and they can be visualized using computed tomography (CT) or magnetic resonance imaging (MRI) (Bernbaum et al., 2015). Existing research recognized a critical role played by WML in several neurological diseases, including cognitive impairment, dementia (Prins and Scheltens, 2015), and Alzheimer's disease (AD) (Van Den Berg et al., 2018). It has also previously been observed that WML can increase the risk of HT in patients with ischemic stroke (D'anna et al., 2021). In recent meta-analyses, WML and its severity were proven to be related to HT and sICH after IVT treatment in patients with stroke (Charidimou et al., 2016; Lin et al., 2016). An increase in the severity of WML was found to indicate a high risk of sICH after reperfusion (Rastogi et al., 2022). However, no current review has explored the difference between the distribution of WMLs, for example, deep WML (DWML), and periventricular WML (PWML), and their association with HT. In this meta-analysis and systematic review, we aimed to comprehensively evaluate and summarize the association between WML and HT in patients with ischemic stroke, taking both the severity and distribution of WML into account.

## Materials and methods

### Search strategy

This meta-analysis was prepared with reference to the Preferred Reporting Items for Systematic Reviews and Meta-Analyses (PRISMA) (Moher et al., 2009) and was conducted according to the prespecified protocol (PROSPERO registration number: CRD42022313467) designed in October 2021.

We searched PubMed, Web of Science, Embase, and Cochrane Library databases from inception to 3 November 2021 for studies on WML and HT in patients with different types of ischemic stroke. Search terms included “leukoaraiosis” or “white matter lesion” or “white matter hyperintensity” or “white matter disease” and “hemorrhage transformation” or “intracerebral hemorrhage” or “intracranial hemorrhage.” Full search terms used in this research are shown in the Supplementary material.

### Inclusion and exclusion criteria

Studies were included for the meta-analysis if they met the following criteria: (1) original publications; (2) specific definitions of WML and HT; (3) studies that included ischemic stroke patients with WML; and (4) HT and/or subtypes of HT described as outcomes. The exclusion criteria were given as follows: (1) duplicate publications or duplicate sets of patients in different studies; (2) animal studies; (3) abstracts or meetings; (4) systematic review and/or meta-analysis; (5) risk estimates did not provide directly and could not construct a 2 × 2 table; (6) no assessment of relevant outcomes; and (7) WML not at baseline. During the process of screening eligible research, the reviewers were blinded to the authors and institution of the studies being reviewed.

### Quality assessment

The quality of included studies was assessed based on the Newcastle–Ottawa Scale (NOS) (Stang, 2010), a tool for assessing the risk of bias in observational studies. An article was considered to be of high quality when the NOS score was equal to or more than 6. Two authors independently assessed the quality of each study. The quality assessment results are shown in Supplementary Table S2.

### Data extraction

Two authors independently extracted data from all potentially relevant articles, and any disagreements were resolved through discussion with a third author. The following information was extracted from eligible studies through a data extraction sheet: author's name, year of publication, country, study design, sample size, age, gender, stroke type based on Trial of Org 10172 in acute stroke treatment (TOAST) classification, whether ischemic stroke secondary to large vessel occlusion (LVO) or not, whether anterior circulation stroke or not, radiology, grading scale for WML, the definition of HT, treatment, sample size, number of cases and control, effect estimates and confidence intervals for every outcome.

As for data about WML, the presence, severity, and distribution of WML were taken into account. Considering the varied subtypes of HT, including PH, HI, sICH, and remote ICH (rICH) in patients with ischemic stroke, these subtypes were regarded as different outcomes in our analysis of the severity of WML and the risk of HT. However, the specific subtype of HT was not considered for articles with the distribution of WML, as the number of these articles was relatively small.

### Statistical analysis

Data were pooled in a meta-analysis for no <3 articles applying STATA 16.0 and R version 4.0.2. Based on the quantity of included articles, we finally conducted seven groups of meta-analysis to quantify the strength of the associations between the presence of WML, moderate-to-severe WML, severe WML, and HT, and its different subtypes (sICH and PH). Odds ratios (ORs) and 95%CIs were obtained directly from studies or calculated using the number of cases and control. Heterogeneity was assessed by *I*^2^ and *p*. *P-*values < 0.05 were considered statistically significant. The random-effects model with DerSimonian–Laird weights were adopted to quantify the strength of the association between the severity of WML and HT using ORs and 95%CIs. Subgroup analyses were conducted to explore the potential confounding factors, including country, study design (prospective/retrospective), stroke type (TOAST classification; ischemic stroke secondary to large vessel occlusion (LVO) or not; anterior circulation stroke or not), treatment (IVT/EVT), and the imaging used for evaluating WML (CT and/or MRI). In addition, the funnel plot with Egger's test was adopted to assess the publication bias. A sensitivity analysis was also conducted to test the contribution of each study to the pool results.

A dose–response meta-analysis was conducted for studies presenting WML analyses in ≥3 levels of severity of WML. To assign “doses” to every WML category, WML measurements using different rating scales were uniformly classified into a 0–3 severity scale (none, mild, moderate, and severe) based on the median WML severity score for each category. We pooled the risk estimates using generalized least squares regression (GLST) with the restricted cubic spline models and predefined knots at the 10th, 50th, and 90th percentile. *P*-values < 0.05 were considered statistically significant.

## Results

### Results of included research

**Figure 1:**
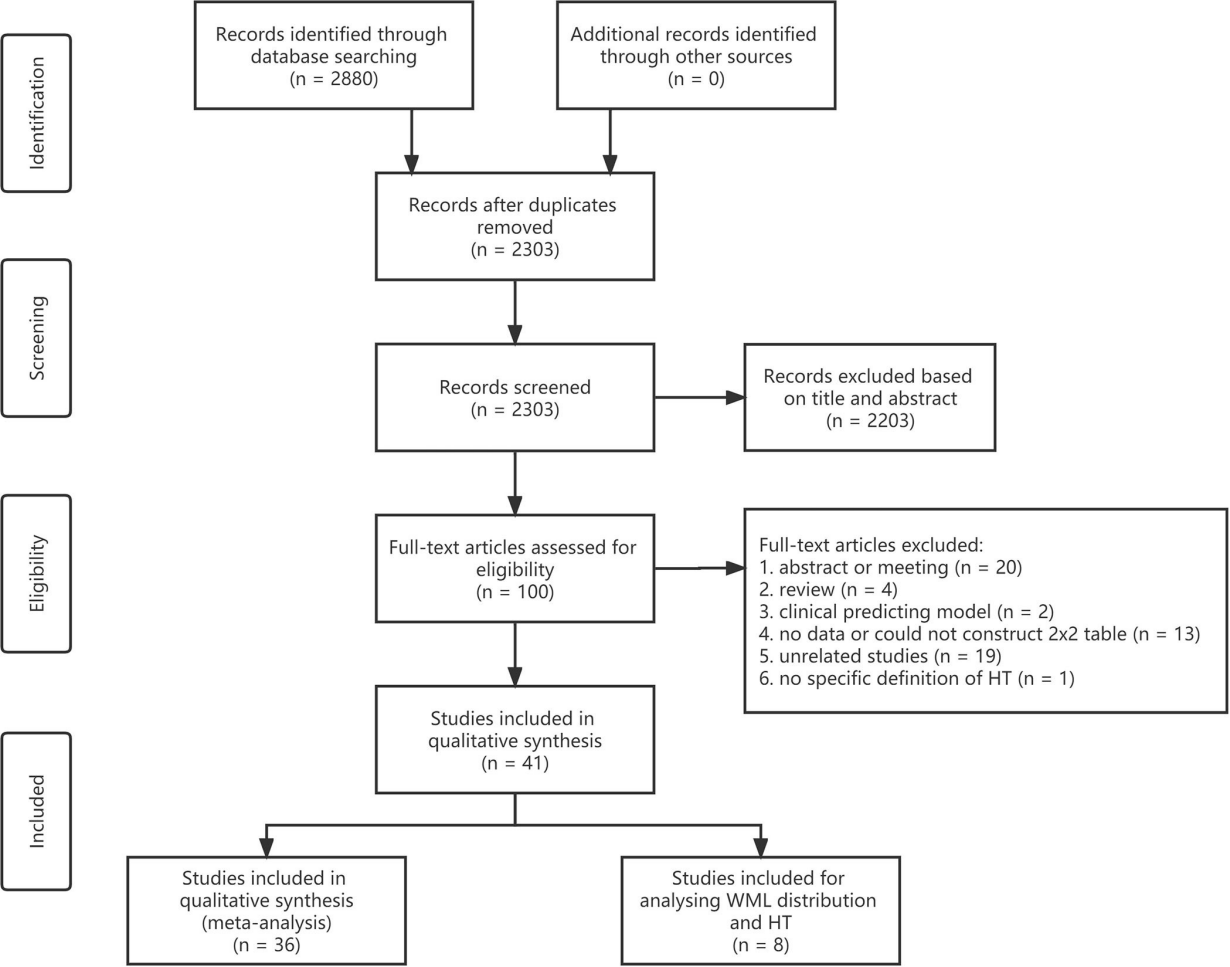
Flowchart of the literature search and study selection. WML, white matter lesion; HT, hemorrhagic transformation.

A total of 2,303 articles were identified after removing duplicates through database searching. Approximately 41 articles met the inclusion criteria and were finally included in this meta-analysis and systematic review (Figure 1) (Rodríguez-Yáñez et al., 2006; Palumbo et al., 2007; Demchuk et al., 2008; Singer et al., 2008; Fiehler et al., 2009; Ariës et al., 2010; Choi et al., 2011; Cho et al., 2012; Costello et al., 2012; Jung et al., 2012; Kawano et al., 2012; Shi et al., 2012; Zheng et al., 2012; Kufner et al., 2013; Curtze et al., 2015, 2016; Wardlaw et al., 2015; Willer et al., 2015; Prats-Sanchez et al., 2017; Wei et al., 2017, 2019; Chen et al., 2018; Liu et al., 2018, 2019; Yang et al., 2018; El Nawar et al., 2019; Guo et al., 2019; Delcourt et al., 2020; Drelon et al., 2020; Eryildiz et al., 2020; Mistry et al., 2020; Mutzenbach et al., 2020; Tanaka et al., 2020; Luijten et al., 2021; Qiu et al., 2021; Albo et al., 2021; Benson et al., 2021; D'anna et al., 2021; Brainer Clares De Andrade et al., 2022; Wang et al., 2022). Approximately 36 articles were adopted in analyzing the association between the severity of WML and HT, seven groups of meta-analyses were conducted, and eight articles were adopted in analyzing the distribution of WML and HT. One article can be used in different groups of meta-analysis and descriptive analysis as it may present data on different grades or distributions of WML as an intervention and different types of HT as the outcome.

Detailed baseline characteristics of all included studies for meta-analysis are shown in Supplementary Table S1. The subtype of ischemic stroke based on the TOAST classification mentioned was a cardioembolic stroke. IVT treatment was adopted as at least one type of treatment in the majority of articles (25/36, 69.4%). The grading scale used for WML included the Fazekas Scale score (Fazekas et al., 1987), the Van Swieten (VSS) Scale score (Van Swieten et al., 1990), modified VSS (mVSS) score (Palumbo et al., 2007), the modified rating scale based on Automated software-based Alberta Stroke Program Early CT Score (ASPECTS) (Barber et al., 2000), and age-related white matter changes (ARWMC) (Wahlund et al., 2001). We classified the severity of WML according to the corresponding grading scale if it was not mentioned in the articles directly. For example, the Fazekas Scale scores of 1–2, 3–4, and 5–6 were classified as mild, moderate, and severe WML, respectively, with a score ranging from 0 to 6 (Fazekas et al., 1987).

### The severity of WML and HT after ischemic stroke

**Figure 2:**
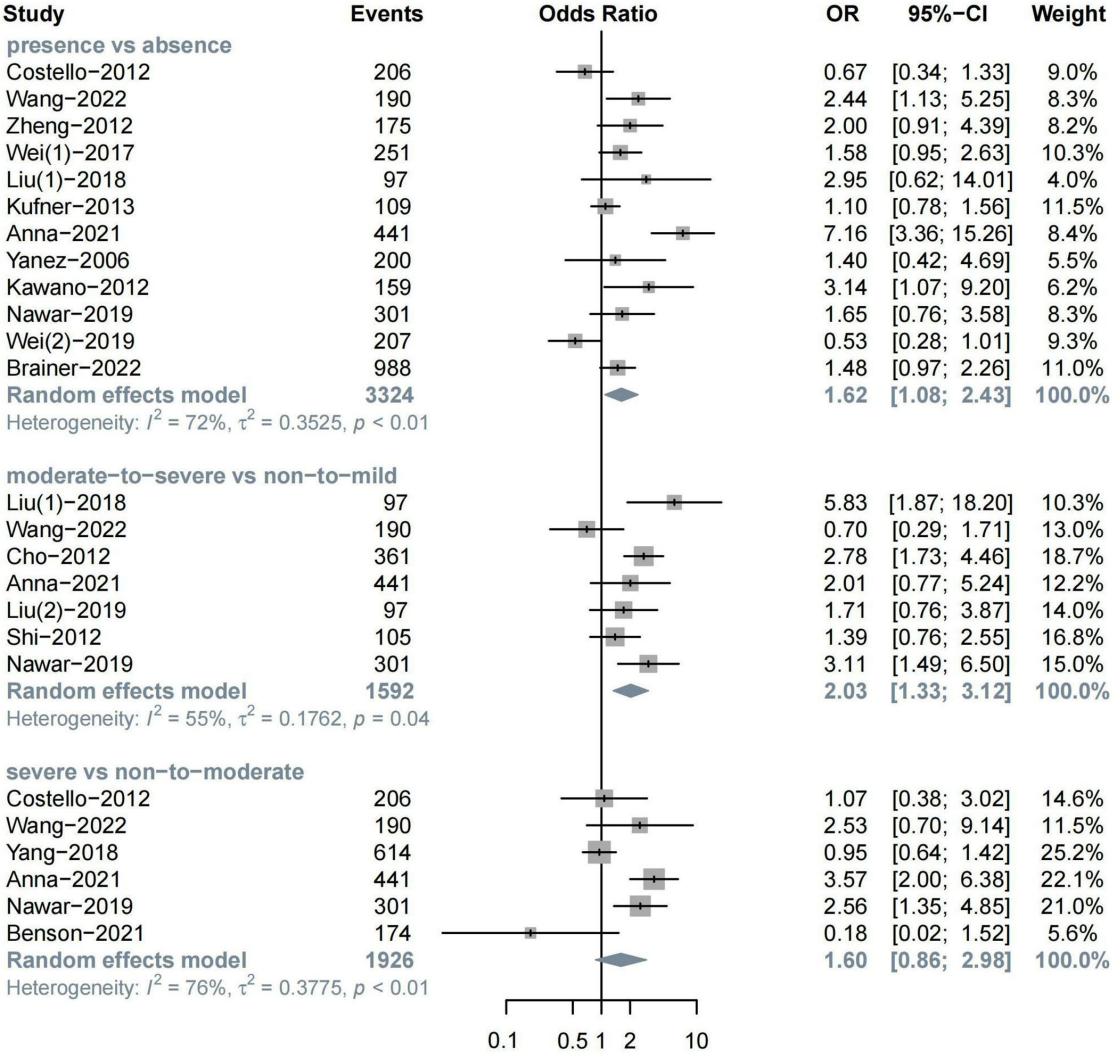
The severity of WML and the risk of HT.

The meta-analysis of 12 studies on HT after ischemic stroke showed that the presence of WML indicated a higher risk of HT (*n* = 12, OR = 1.62, 95%CI 1.08–2.43, *p* = 0.019). In addition, moderate-to-severe WML showed a more significant association with HT (*n* = 7, OR = 2.03, 95%CI 1.33–3.12, *p* = 0.001). The association between severe WML and HT did not reach the traditional level of statistical significance (*n* = 6, OR = 1.60, 95%CI 0.86–2.98, *p* = 0.136) (Figure 2).

In MRI subgroups, both the presence (*n* = 6, OR = 2.14, 95%CI 1.22–3.77) and severity of WML (*n* = 4, OR = 2.69, 95%CI 1.77–4.11) could increase the risk of HT over two times, while no significant association was found in the CT group. In the treatment subgroup, the presence of WML was found to be related to spontaneous HT (*n* = 3, OR = 1.64, 95%CI 1.15–2.33), which is defined as IVT, and EVT was not administered in this study (Alexandrov et al., 1997; Tan et al., 2014) (Supplementary Figures S1–S3).

### The severity of WML and sICH after ischemic stroke

**Figure 3:**
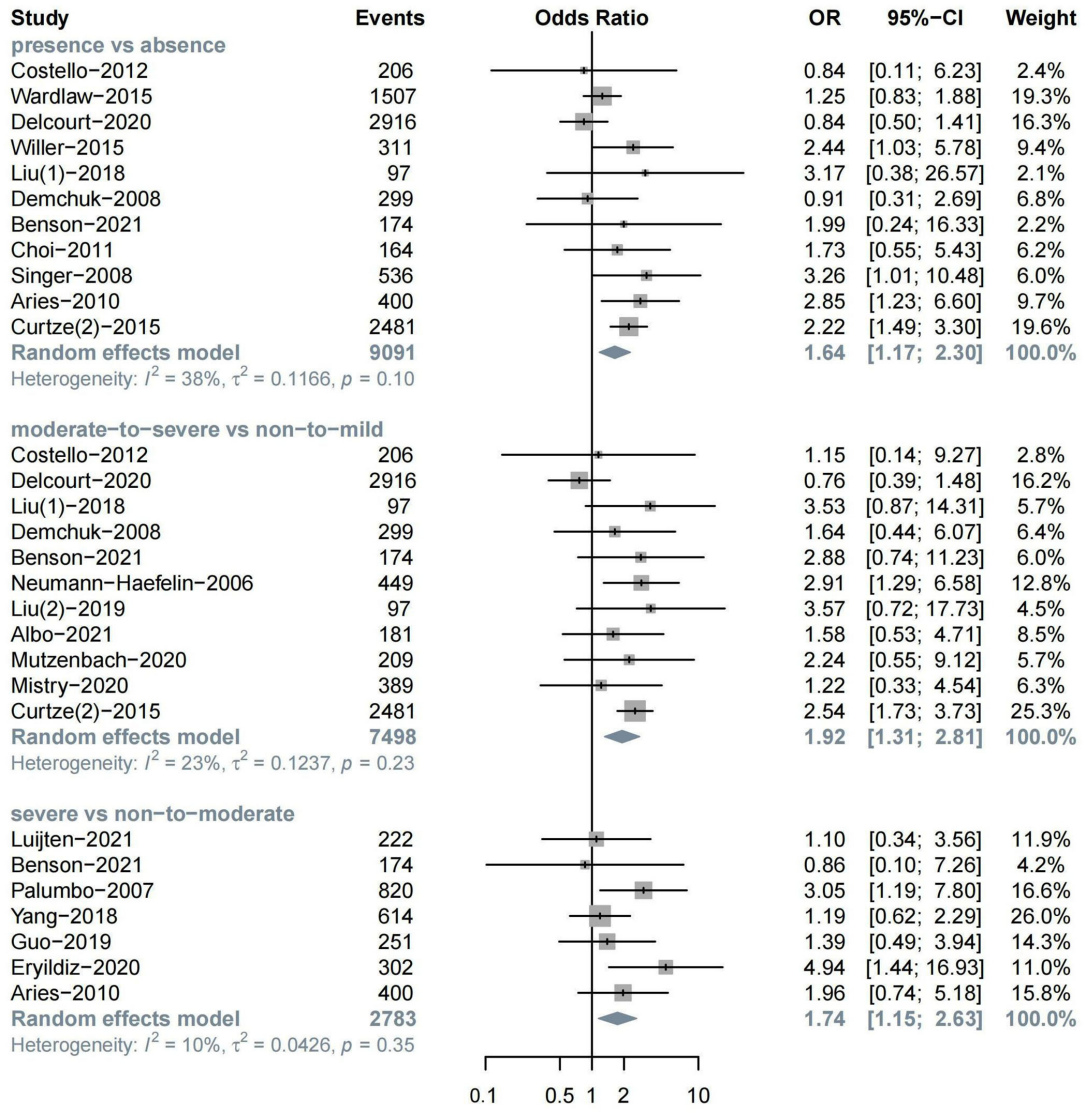
The severity of WML and risk of symptomatic intracerebral hemorrhage (sICH).

The meta-analysis of 11 studies about sICH after ischemic stroke showed that WML was associated with sICH (*n* = 11, OR = 1.64, 95%CI 1.17–2.30, *p* = 0.004). Evidence showed that moderate-to-severe WML was associated with a high risk of sICH (*n* = 11, OR = 1.92, 95%CI 1.31–2.81, *p* < 0.001). Severe WML could also increase the risk of sICH (*n* = 7, OR = 1.74, 95%CI 1.15–2.63, *p* = 0.009) (Figure 3).

In the subgroup analysis, we found a significant association between moderate-to-severe WML and sICH in both the IVT treatment (*n* = 6, OR = 1.86, 95%CI 1.07–3.24) and EVT treatment groups (*n* = 5, OR = 2.00, 95%CI 1.11–3.61). In addition, moderate-to-severe WML was associated with a relatively higher risk of sICH in patients with the anterior circulation stroke (*n* = 4, OR = 2.41, 95%CI 1.35–4.31) (Supplementary Figures S4–S6).

### Severity of WML and other subtypes of HT

**Figure 4:**
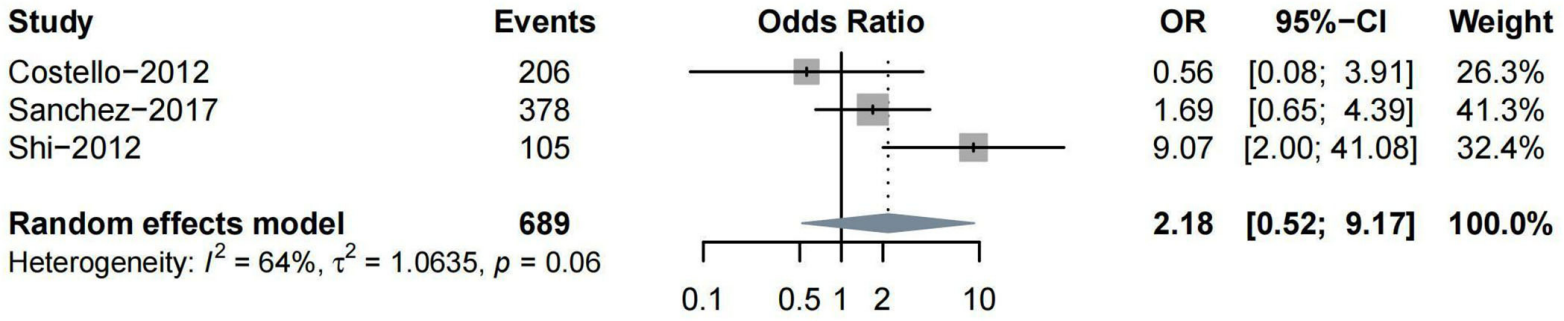
The severity of WML and the risk of parenchymal hematoma (PH).

Other subtypes of HT included PH, HI, and rICH. Four studies reported PH as one of the outcomes. Three (Costello et al., 2012; Shi et al., 2012; Prats-Sanchez et al., 2017) studies reported ORs on moderate-to-severe vs. non-to-mild WML, while only one (Curtze et al., 2016) study classified the severity of WML into severe vs. non-to-moderate. Thus, we conducted a meta-analysis for the association between moderate-to-severe WML and PH. No significant association was found between them (*n* = 3, OR = 2.18, 95%CI 0.52–9.17, *p* = 0.288) (Figure 4).

Two studies analyzed the association between WML and HI, and no significant association was found (Costello et al., 2012; Nighoghossian et al., 2016). Only one study reported the association between mild WML and rICH after IVT (Drelon et al., 2020). Due to insufficient data, a meta-analysis could not be performed to quantify their relationship.

### Distribution of WML and HT after ischemic stroke

Eight studies provided data on the relation between WML distribution and HT after ischemic stroke (Table 1). Due to the heterogeneity in WML data presentation (either as rating score, volume in ml, or percentage of intracerebral volume), these studies could not be pooled for meta-analysis.

**Table 1:** Relation between the distribution of white matter lesion (WML) and hemorrhagic transformation (HT).

**Study**	**Sample**	**WMH localization**	**Results**	**OR and 95%CI or other summary measure**	**Comment**
Wei et al. (2017)	251	DWMH and PWMH rating	DWML+	PVH > 1: 2.850 (1.322–6.143)	The presence of periventricular or anterior LA on MRI
			PWML+	DWMH > 0: 2.029 (1.021–4.029)	increases the risk of HT
Neumann-Haefelin et al. (2006)	363	DWMH and PWMH rating	DWML+	8 DWML patients (8 in 62) had sICH	LA of the DWM is the important aspect conferring an increased risk for sICH
			PWML–	0 PWML patient (0 in 27) had sICH	
Shi et al. (2012)	105	DWMH and PWMH rating	DWML+ PWML–	DWML: 3.43 (1.23–9.57)	Patients with only periventricular LA did not have a higher rate of parenchymal hematoma. Moderate or severe LA in the DWM was an independent predictor of hemorrhagic transformation
Fiehler et al. (2009)	100	DWMH and PWMH rating	DWML–	DWML: sensitivity: 0.41 specificity: 0.81	The DWML score and the sum of both ratings were not substantially more predictive; the PVML rating seems
			PWML+	PWML: sensitivity: 0.86 specificity: 0.65	to be sufficient in order to save time in the acute situation
Chen et al. (2018)	503	DWMH and PWMH	DWML+	DWML: 1.868 (1.142–3.056)	Not only the corrected deep periventricular
		volume		(per 10 ml)	white matter hyperintensity volume, but the corrected
			PWML+	PWML: 1.912 (1.320–2.770) (per 10 ml)	deep white hyperintensity volume was associated with the occurrence of rICH
Jung et al. (2012)	292	PWMH rating	PWML+	PWML: sICH: 0.92 (mean Fazekas score) No sICH: 0.89 (mean Fazekas score)	Both the Fazekas and the Scheltens scores revealed white matter lesions in the periventricular region to be most predictive for clinical outcome and survival
Tanaka et al. (2020)	455	DWI-ASPECTS + W	DWML+	DWI-ASPECTS + W: *p* < 0.001	DWI-ASPECTS + W predicted sICH more accurately than CT-ASPECTS and DWI-ASPECTS in patients who received intravenous rt-PA for acute ischemic stroke.
Qiu et al. (2021)	254	Periventricular TPP and subcortical TPP	Periventricular TTP+	Length of periventricular TTP: 4.4740 (1.624–13.837)	Chronic white matter hypoperfusion was independently associated with intracranial hemorrhage after thrombolysis. The best discriminating value of periventricular TTP was also determined.

+, relation between WML distribution and ICH; –, no relation between WHM distribution and ICH.

PVH, periventricular hyperintensity; PWMH, periventricular white matter hyperintensity; DWMH, deep white matter hyperintensity; DWI-ASPECTS + W, DWI-ASPECTS including early ischemic changes in deep white matter; LA, leukoaraiosis; TTP, transit time to the peak.

Of these eight studies, five distinguished articles discussed about PWML and DWML and the other three articles reported the relation between either PWML or DWML alone and HT. Of the five studies distinguishing PWML and DWML, three showed the results that PWML was more efficient in predicting HT, while the other two studies concluded that patients with ischemic stroke complicated with PWML alone were not at a higher risk of HT. Both PWML and DWML were found to be related to HT in the majority of studies (5/7 and 5/6, respectively).

### Publication bias and sensitivity analysis

The funnel plot did not show obvious publication bias (Supplementary Figure S7). We also conducted Egger's test to quantify publication bias, and no significant bias was found (*p* > 0.05) (Supplementary Figure S8). A sensitivity analysis showed that the results of the meta-analyses in our study were stable, as the conclusion of our study did not change after included studies were excluded, with the exception of studies of Benson et al. (2021) or Yang et al. (2018) in the severity of WML and the risk of HT groups (Supplementary Figure S9).

### Dose–response associations between WML and HT/sICH

**Figure 5:**
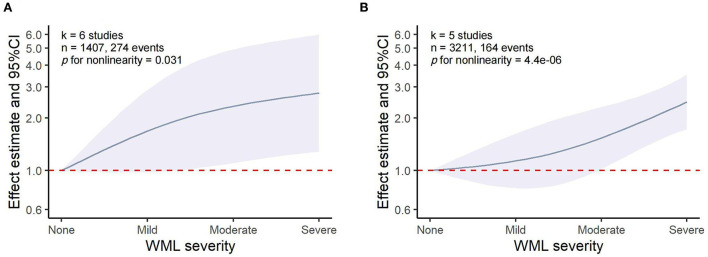
Dose–response meta-analysis of the association between WML and HT/sICH. **(A)** WML severity and HT. **(B)** WML severity and sICH.

The dose–response meta-analysis showed a non-linear association between WML and both HT (*p* for non-linearity = 0.031, six studies, 1,407 individuals, and 274 events) and sICH (*p* for non-linearity = 4.4 × 10^−6^, five studies, 3,211 individuals, and 164 events). Specifically, it was found that WML increased the risk of HT and sICH above a moderate severity, which was consistent with the results of our meta-analysis that moderate-to-severe WML was significantly associated with an increased risk of both HT and sICH (Figure 5). However, these results should be interpreted with caution as the pooled relative risk (RR) effect in the dose–response analysis was unadjusted.

## Discussion

This systematic review and meta-analysis summarized current evidence on the association between WML and HT. The results showed the association between WML and HT in patients with ischemic stroke, and as expected, increasing the severity of WML was found to be associated with a high risk of both HT and sICH. Our descriptive analysis suggested that both PWML and DWML could increase the risk of HT.

White matter lesions, a common type of CSVDs potentially found on neuroimaging, represent abnormal focal lesions of brain white matter (Sam et al., 2016). Previous reports identified an association between the presence and severity of WML and HT in patients with ischemic stroke. Wei et al. (2017) reported WML as an independent risk factor for HT after cardioembolic stroke based on the atrial fibrillation (AF) and/or rheumatic heart disease (RHD) population. In the third International Stroke Trial (IST-3), pre-existing WML was found to increase the risk of sICH (Wardlaw et al., 2015). In addition to sICH, the increased risk of other subtypes of HT in patients with stroke complicated with WML was also mentioned. Nighoghossian et al. (2016) retrospectively analyzed the association between WML and PH in elderly patients and presented a positive result, and this result was in accordance with Shi et al. (2012) who found that the rate of PH was two times as high in patients with moderate-to-severe LA than in those without. Pre-existing brain lesions including WML were also found to help predict rICH (Drelon et al., 2020). However, some reports demonstrated the opposite results. For example, a study based on 206 patients with ischemic stroke reported that WML did not increase the risk of HT including PH after thrombolysis (Costello et al., 2012). To clarify whether WML at baseline might indicate a high risk of HT in patients with ischemic stroke, especially after IVT treatment, several meta-analyses were already conducted. The presence of WML may increase the risk of post-thrombolysis intracerebral hemorrhage in patients with ischemic stroke (Lin et al., 2016). However, Lin et al. (2016) failed to classify overall HT and sICH as different outcomes. Another meta-analysis concluded that the presence of WML might increase the risk of post-IVT sICH (Charidimou et al., 2016). The importance of the severity of WML was also noted by prior systematic reviews. Kongbunkiat et al. (2017) reported that both the presence and greater severity of WML were related to a high risk of sICH. Nevertheless, the severity of WML did not influence the incidence of sICH in patients with acute ischemic stroke administered with EVT (Huo et al., 2020). A recent study exploring the association between the severity of WML and post-reperfusion outcomes including HT with subgroup analysis for the treatment of patients (IVT and/or EVT) suggested the efficiency of moderate-to-severe WML on the baseline as a predictor for sICH, especially after IVT in acute ischemic stroke (Rastogi et al., 2022). Our findings, which show a correlation between the presence of both WML and moderate-to-severe WML and an increased risk of HT and sICH, is consistent with the results of these meta-analyses. According to our meta-analysis and the dose–response analysis, patients with ischemic stroke are at a higher risk of HT and sICH in anticipation of an increase in the severity of WML. One unexpected finding is a weak association of severe WML with HT. It is likely due to the already increased risk of HT in patients with moderate WML as we conducted the meta-analysis of binary data (severe WML vs. non-to-moderate WML). This explanation is consistent with the result of the dose–response analysis, which showed that the risk of HT increased relatively slightly from moderate WML to severe WML. Notably, data from Benson et al. (2021) had a significant influence on a weak association between severe WML and HT as the incidence of HT was unexpectedly the lowest in patients with severe WML in a retrospective study. The anomaly might suffer from inadequate sample size. In addition, we propose that the grading scale for the severity of WML was also a possible confounding factor as Benson et al. (2021) used the Leukoaraiosis-ASPECTS (L-ASPECTS) scale based on CT rather than the traditional Fazekas Scale based on MRI.

In our subgroup analysis, the type of treatments adopted in patients with ischemic stroke was a dominant variable. A broadly significant association between WML and the risk of HT was found in the IVT group, and the result that moderate-to-severe WML may be a risk factor for HT in patients with EVT, which is in agreement with that obtained by Rastogi et al. (2022). Previous studies demonstrated that moderate-to-severe WML might increase the risk of sICH after IVT (Charidimou et al., 2016; Lin et al., 2016), which is consistent with the current findings. We also found a more prominent association between moderate-to-severe WML and sICH in the EVT treatment subgroup, which is consistent with a previous study that found deep white matter on diffusion-weighted imaging (DWI) to be a predictor of sICH after EVT (Hao et al., 2019). Nevertheless, this finding contradicts a recent study that found moderate-to-severe WML to be associated with sICH only in patients receiving IVT rather than EVT (Rastogi et al., 2022). As mechanical thrombectomy (MT) has become the standard treatment in acute ischemic stroke secondary to a LVO, the majority of the studies in our meta-analysis that adopt EVT as the treatment were based on the LVO population. The incidence of sICH was reported to be higher with EVT in LVO ischemic stroke than with IVT in acute ischemic stroke according to previous clinical trials (Hacke et al., 2008; Goyal et al., 2016). Therefore, it could conceivably be explained that EVT treatment already indicated a higher risk of intracerebral hemorrhage than IVT in our meta-analysis. In addition, the results obtained from Huo et al. (2020) that severe WML was not associated with sICH after EVT differed from the findings presented here. Another possible explanation for the inconsistency with the result of the prior meta-analysis is that certain research classifying WML into VSS ≤ 4 and VSS > 4 (Palumbo et al., 2007; Guo et al., 2019; Luijten et al., 2021) were pooled in the severe WML vs. non-to-moderate group in our study, while the research of Rastogi et al. (2022) only dichotomized the severity of WML into moderate-to-severe WML vs. non-to-mild WML. The presence of WML was found to increase the risk of spontaneous HT. This result has been unable to demonstrate that the risk of post-antithrombotic HT is higher than that of spontaneous HT, and it can possibly be due to the relatively small number of studies on spontaneous HT pooled in the meta-analysis and the lack of quantification of the severity of WML. However, these findings in the subgroup analysis cannot determine whether patients with ischemic stroke complicated with the corresponding severity of WML can benefit from IVT or EVT due to the lack of patients in which IVT or EVT is withheld. In addition to treatments, imaging used in studies was also a possible confounding factor. A previous meta-analysis reported that the association between WML and the outcomes of intracerebral hemorrhage was significant in the CT-assessed subgroup rather than the MRI (Yu et al., 2019). However, in our subgroup analysis, both the presence and severity of WML were found to increase the risk of HT in the MRI-assessed group, while no significant association was found in the CT group. This result may be due to the better precision of MRI compared to CT in evaluating WML (Lin et al., 2016). In addition, the risk of sICH was found to be relatively high in the anterior circulation stroke group, which corroborates the findings of previous studies that the incidence of both HT and sICH is higher in the anterior circulation stroke than in the posterior circulation stroke (Sarikaya et al., 2011; Tong et al., 2016).

The exact mechanism of WML impacting HT in patients with stroke is not well understood; however, it is believe that WML may play an indirect role in increasing the risk of HT (Rastogi et al., 2022). Clinical trials suggested that WML contributed to an increased risk of hemorrhage and poor neurological recovery (Smith et al., 2002; Henninger et al., 2012). Thus, endothelial dysfunction and blood–brain barrier (BBB) damage are possible hypothetical mechanisms to explain this association. Endothelial dysfunction is one certain cause of WML as it can cause direct damage to myelin as well as hamper myelin formation and repair by blocking oligodendrocyte cell maturation (Rajani et al., 2018). It can further destroy the completeness of BBB and modify the cerebral tissue, increasing the risk of hemorrhage.

Current reviews about the relation between WML and HT failed to consider the distribution of WML. PWML and DWML are considered to have dissimilar pathogenic mechanisms (Schmidt et al., 2011), and the heterogeneity between DWML and PWML has been reviewed in their association with a cognitive function. For example, Van Den Berg et al. (2018) quantified the association of white matter hyperintensities (WMHs) and cognitive domains in patients with mild cognitive impairment (MCI) or AD and suggested that periventricular WMHs (PWMHs) were more strongly related to cognition impairment than deep WMHs (DWMHs). In our descriptive analysis, the majority of studies making a distinction between PWML and DWML indicated an association between PWML and DWML with HT. Due to the high heterogeneity of included articles, data consolidation using a meta-analysis is not suitable. However, the distinction between PWML and DWML is still arbitrary. In a multicenter analysis of 363 patients, DWML was identified as an independent risk factor for sICH after IVT treatment, while none of the 27 patients with PWML alone experienced sICH (Neumann-Haefelin et al., 2006). The result of the aforementioned study was in line with that of another study, which reported that moderate-to-severe DWML was an independent predictor of HT (Shi et al., 2012). There is still a dearth of prospective clinical trials with large sample sizes focusing on the association between WML in different areas and HT. Interestingly, in a diagnostic experiment, the authors suggested PWML as a more time-saving factor for evaluating the risk of HT compared to DWML as PWML showed higher sensitivity but lower specificity in diagnosing parenchymal hematoma compared to DWML (Fiehler et al., 2009).

The pathological mechanism underlying the differences in the influence of PWML and DWMLs is not yet clear. Based on prior pathological research, PWML may be more hemodynamically derived as the corresponding area is mainly supplied by vessels originating from choroidal arteries or terminal branches of the rami striate that are prone to have an ischemic change, while deep white matter areas fed by medullary arteries are more sensitive to small-vessel disease (SVD) (Gouw et al., 2011; Cai et al., 2022). The possible pathological mechanisms of WML in different areas are also related to the severity of WML. No arteriolosclerotic changes were observed in mild PWML, whereas more severe PWML and DWML show varying myelin loss, reactive gliosis, and incomplete parenchymal destruction (Kim et al., 2008). Therefore, moderate-to-severe WML in both areas is likely to increase the risk of HT in patients with ischemic stroke, especially in the condition that the arteriolar wall damage becomes more difficult to repair after antithrombotic treatment.

Our study has several strengths. First, to our knowledge, this is the first systematic review and meta-analysis to evaluate the association between both the severity and distribution of WML and HT. Specifically, we pooled data on the association between the severity of WML and HT into three groups: the presence of WML vs. non WML; moderate-to-severe WML vs. non-to-mild WML; and severe WML vs. non-to-moderate WML. We also analyzed the risk effects of increasing the severity of WML on HT and sICH through a dose–response meta-analysis. We also reviewed different influences of the distribution of WML in predicting HT. Second, the different subtypes of HT were also considered when analyzing the association between the severity of WML and the risk of HT. Third, the studies included in our study were more comprehensive and had a larger sample size than previous systematic reviews.

We would like to acknowledge the limitations of our study. The analysis of the association between the distribution of WML and HT is not ideal, as, compared to the severity of WML, a smaller number of studies classified WML into DWML and PWML or other appropriate classification. In addition, the measurement data in these articles, including the percentage and volume of WML in different locations, were not qualified to be pooled in a meta-analysis. These articles were reviewed in our descriptive analysis.

Therefore, further studies with a larger sample size on the association between WML and HT and taking the distribution of WML into account are suggested. More outcome measures, including the modified ranking scale (mRS) for functional outcomes, are needed to conduct more comprehensive research on the influence of WML on the prognosis of patients with ischemic stroke. In addition, the continuous effect of WML is also recommended to display the correlation between WML and outcomes more clearly as the severity of WML increases.

In conclusion, this meta-analysis and systematic review demonstrated that patients with ischemic stroke complicated with WML are at an increased risk of HT and sICH, and those with ischemic stroke complicated with more severe WML indicate a relatively high risk of HT and sICH. In addition, both PWML and DWML could be risk factors for predicting HT. These data may help predict HT and its subtypes, which could be severe complications of reperfusion therapy in patients with ischemic stroke complicated with WML clinically, and potentially improve the management of patients with ischemic stroke.

## Data availability statement

The original contributions presented in the study are included in the article/Supplementary material, further inquiries can be directed to the corresponding author.

## Author contributions

YW, XB, CY, and YY study concept, data collection, and writing of the manuscript. YW and XB statistical analysis and manuscript drafting. BW study supervision and critical revision of manuscript. All authors have read and approved the manuscript.

## Funding

This work was supported by the innovation and entrepreneurship training program of Sichuan University (No. C2022121306).

## Conflict of interest

The authors declare that the research was conducted in the absence of any commercial or financial relationships that could be construed as a potential conflict of interest.

## Publisher's note

All claims expressed in this article are solely those of the authors and do not necessarily represent those of their affiliated organizations, or those of the publisher, the editors and the reviewers. Any product that may be evaluated in this article, or claim that may be made by its manufacturer, is not guaranteed or endorsed by the publisher.
