# Supplementary material for: The association between the severity and distribution of white matter lesions and hemorrhagic transformation after ischemic stroke: A systematic review and meta-analysis
Source: Front Aging Neurosci. 2022 Nov 25;14:1053149. doi: 10.3389/fnagi.2022.1053149 (PMC9732368; doi:10.3389/fnagi.2022.1053149)
Supplement: Supplementary file 1 [file Data_Sheet_1.docx]

**Supplementary material**

**List of contents**

1. **Search strategy**
2. **Supplemental Table 1.** Baseline characteristics of included studies
3. **Supplemental Table 2.** Quality assessment
4. **Supplemental Figure 1.** Subgroup analysis of presence of WML and risk of HT
5. **Supplemental Figure 2.** Subgroup analysis of moderate-to-severe WML and risk of HT
6. **Supplemental Figure 3.** Subgroup analysis of severe WML and risk of HT
7. **Supplemental Figure 4.** Subgroup analysis of presence of WML and risk of sICH
8. **Supplemental Figure 5.** Subgroup analysis of moderate-to-severe WML and risk of sICH
9. **Supplemental Figure 6.** Subgroup analysis of severe WML and risk of sICH
10. **Supplemental Figure 7.** Funnel plot of included studies
11. **Supplemental Figure 8.** Egger’s test
12. **Supplemental Figure 9.** Sensitivity analysis of meta-analysis (influence of a single study)
13. **PRISMA 2020 checklist**
14. **Search strategy**

**PubMed (1189)**

("WMH" OR "WML" OR "LA" OR "white matter lesion*" OR "white matter hyperintensit*" OR "white matter disease*" OR "white matter change*" OR "leukoaraiosis") AND ("ICH" OR "sICH" OR "HT" OR "brain hemorrhage" OR "intracranial hemorrhage" OR "Intracerebral hemorrhage" OR "cerebral hemorrhage" OR "symptomatic intracranial hemorrhage" OR "symptomatic intracerebral hemorrhage" OR "hemorrhagic transformation") AND (stroke OR "cerebral infarction" OR "brain infarction" OR "ischemic stroke")

**Embase (668) Adcanced Search, no related terms**

('white matter lesion'/exp OR 'white matter hyperintensity'/exp OR 'white matter hyperintensities'/exp OR 'white matter disease'/exp OR 'white matter change'/exp OR 'leukoaraiosis'/exp) AND ('brain hemorrhage'/exp OR 'intracranial hemorrhage'/exp OR 'intracerebral hemorrhage'/exp OR 'cerebral hemorrhage'/exp OR 'symptomatic intracranial hemorrhage'/exp OR 'symptomatic intracerebral hemorrhage'/exp OR 'hemorrhagic transformation'/exp) AND ('acute ischemic stroke'/exp OR 'brain infarction'/exp OR 'cerebral infarction'/exp OR 'stroke'/exp)

**Web of Science (1023)**

((TS=“WMH”) OR (TS=“WML”) OR (TS=“LA”) OR (TS=“white matter lesion*”) OR (TS=“white matter hyperintensit*”) OR (TS=“white matter disease*”) OR (TS=“white matter change*”) OR (TS=“leukoareosis”) OR (TS=“leukodystrophy”)) AND ((TS=“ICH”) OR (TS=“sICH”) OR (TS=“HT”) OR (TS=“brain hemorrhage”) OR (TS=“intracranial hemorrhage”) OR (TS=“Intracerebral hemorrhage”) OR (TS=“cerebral hemorrhage”) OR (TS=“symptomatic intracranial hemorrhage”) OR (TS=“symptomatic intracerebral hemorrhage”) OR (TS=“hemorrhagic transformation”)) AND ((TS=“stroke” ) OR (TS=“cerebral infarction”) OR (TS=“brain infarction”) OR (TS=“ischemic stroke”))

**Cochrane Library (136)**

#1 (white matter hyperintensity) OR (white matter hyperintensities) OR (white matter lesion) OR (white matter disease) OR (leukoaraiosis) (Word variations have been searched) 1538

#2 (intracranial hemorrhage) OR (brain hemorrhage) OR (hemorrhagic transformation) OR (intracerebral hemorrhage) OR (cerebral hemorrhage) (Word variations have been searched) 10996

#3 (ischemic stroke) OR (brain infarction) OR (cerebral infarction) OR (stroke) (Word variations have been searched) 76466

#4 #1 and #2 and #3 136

1. **Supplemental Table 1.** Baseline characteristics of included studies

| Author | Year | Country | Study design | Participants | Male(%) | Age(years) | Stroke Type (TOAST) | Large vessel occlusion (LVO) or not | anterior circulation stroke or not | Initial Stroke Severity (NIHSS) | Imaging for WML | LA Grading | Imaging for HT | HT definition and/or classification | Treatment |
| --- | --- | --- | --- | --- | --- | --- | --- | --- | --- | --- | --- | --- | --- | --- | --- |
| Costello | 2012 | Australia | retrospective | 206 | 54.9 | 52.085 | AIS | Not | Not | 13 | CT | mVSS severe: mVSS > 4 | CT/MRI(T2) | HT: ECASS criteria sICH: SITS-MOST definition | IVT |
| Zheng | 2012 | China | retrospective | 175 | 60 | NA | AIS | Not | Not | 15 | CT | NA | CT/MRI | HT: ECASS II criteria sICH: NINDS definition | IVT |
| Wardlaw | 2015 | UK | prospective | 1507 | NA | NA | AIS | Not | Not | NA | CT/MRI | Fazekas(for MRI) | CT/MRI | sICH: IST-3 definition | IVT |
| Wei(1) | 2017 | China | prospective | 251 | 39.4 | 68.49(12.43) | cardioembolic stroke | Not | Not | 7 | MRI(FLAIR/T2) | Fazekas/VSS/ARWMC | CT/MRI | HT: hemorrhage within the infarct territory or parenchyma hemorrhage outside the infarct zone that was present on a second CT or MRI | IVT/AC/AP |
| Luijten | 2021 | Netherlands | retrospective | 473 | 59 | 66(55-76) | AIS | LVO | anterior circulation stroke | 17(14-21) | CT | VSS absent to moderate: VSS 0-2 severe: VSS 3-4 | CT | sICH: an increase of 4 points or more on the NIHSS and evidence of intracranial hemorrhage on neuroimaging | EVT±IVT |
| Delcourt | 2020 | Australia | prospective | 2916 | NA | 67(13) | AIS | Not | Not | 8(5-14) | CT/MRI | NA | CT/MRI | sICH: IST3 definition | IVT |
| Curtze(1) | 2016 | Finland | retrospective | 2482 | NA | NA | AIS | Not | Not | NA | CT | Blennow severe: Blennow 5-6 non to moderate: Blennow 0-4 | CT | rPH, iPH: ECASS I criteria | IVT |
| Willer | 2015 | Denmark | retrospective | 311 | NA | 68.03 | AIS | Not | Not | 7 | CT/MRI | ARWMC | CT | sICH: hemorrhage and deterioration of NIHSS of 4 or greater within 36 hours from symptom onset | IVT |
| Liu(1) | 2018 | China | retrospective | 97 | 70.1 | 66.6(9.1) | AIS | Not | Not | 11.1(±5.8) | MRI(DWI/FLAIR) | Fazekas | CT | sICH: ECASS II criteria | IVT |
| Cho | 2012 | Korea | retrospective | 361 | 63.4 | 67 | AIS | Not | Not | 6 | MRI(DWI/FLAIR/GRE/magnetic resonance angiography) | Fazekas mild: Fazekas 0-1 severe: Fazekas 2-3 | MRI | HT: ECASS II criteria | IVT |
| Kufner | 2013 | Germany | retrospective | 109 | 49.5 | 71 | AIS | Not | Not | 7 | MRI(DWI/FLAIR) | Wahlund | MRI(T2) | HT: ECASS I criteria HI: petechial HT without mass effect PH: HT with mass effect | IVT |
| Anna | 2021 | UK | retrospective | 441 | 53.1 | 77 | cardioembolic stroke | Not | Not | 6 | MRI(DWI/ADC/FLAIR/T2) | Fazekas | MRI(GRE/T1/T2) | HT: ECASS criteria | IVT/EVT |
| Demchuk | 2008 | USA | retrospective | 299 | NA | NA | AIS | Not | Not | NA | CT | VSS mild to moderate: VSS 1-2 severe: VSS 3-4 | MRI(T2) | sICH: NINDS definition | IVT |
| Benson | 2021 | USA | retrospective | 174 | 48.8 | 68.0(9.1) | AIS | Not | Not | CT | MRI | Fazekas/ASPECTS mild: 1-2 moderate: 3-4 severe: 5-6 | CT | sICH: ECASS II criteria | EVT |
| Palumbo | 2007 | Italy | retrospective | 820 | 42.76 | 69.99 | AIS | Not | Not | 14 | CT | VSS severe: VSS > 4 | CT/MRI | sICH: any neurologic deterioration judged by the treating physician to be secondary to a new brain hemorrhage as shown in a head CT or MRI | IVT |
| Yang | 2018 | China | retrospective | 614 | 61.9 | 67.3 | AIS | Not | Not | 12 | CT | mVSS severe: mVSS > 4 | CT/MRI | sICH: NINDS definition | IVT |
| Neumann-Haefelin | 2006 | France | retrospective | 449 | NA | 64(14) | AIS | Not | anterior circulation stroke | 13.2(6.1) | MRI(T2) | Fazekas and Schmidt non to mild: 0-1 moderate to severe: 2-3 | CT/MRI | sICH: NINDS definition | IVT |
| Liu(2) | 2019 | China | retrospective | 97 | 61.9 | 70.0(12.4) | AIS | LVO | anterior circulation stroke | 13(9-17) | MRI(FLAIR/DWI/MRA) | Fazekas absent: Fazekas 0 mild: Fazekas 1-2 moderate: Fazekas 3-4 severe: Fazekas 5-6 | CT | sICH: any sign of hemorrhage on follow-up head CT within 24 h combined with clinical deterioration of 4 points or more on the NIHSS score | EVT |
| Choi | 2011 | Korea | retrospective | 164 | 64.6 | 63.64 | AIS | Not | Not | 12.7 | MRI | Fazekas absent to moderate: Fazekas 0-2 severe: Fazekas 3-4 | CT/MRI | sICH: ECASS II criteria | IVT |
| Shi | 2012 | China | retrospective | 105 | 41 | 61.6(19.1) | AIS | Not | anterior circulation stroke | 18.1(6.1) | MRI(DWI/T2) | Fazekas non to mild: 0-1 moderate to severe: 2-3 | CT | HT: ECASS II criteria | EVT |
| Guo | 2019 | China | retrospective | 251 | 62.2 | 64.4(11.8) | AIS | LVO | anterior circulation stroke | 16(12-20) | CT | VSS | CT/MRI | sICH: Heidelberg Bleeding Classification | EVT |
| Yanez | 2006 | Spain | prospective | 200 | 51.5 | 71.9 | AIS | Not | Not | 13 | CT | NA | CT | HT: ECASS II criteria | None |
| Kawano | 2012 | Japan | retrospective | 159 | 55.3 | 75.4 | AIS | Not | Not | 14 | CT/MRI(DWI) | ASPECTS | CT | HT: ECASS I criteria | IVT |
| Nawar | 2019 | France | prospective | 301 | 51.8 | 75 | AIS | Not | Not | 9 | MRI(DWI/ADC/FLAIR/T2) | Fazekas | CT/MRI | HT: ECASS II criteria | IVT |
| Wei(2) | 2019 | China | retrospective | 207 | 58.9 | 67.79 | cardioembolic stroke | Not | Not | 8 | CT/MRI(T1/T2/FLAIR/SWI) | Fazekas | CT/MRI | HT: ECASS II criteria | IVT/AC/AP |
| Albo | 2021 | USA | retrospective | 181 | 45.9 | 68(57-81) | AIS | LVO | Not | 18 | CT | VSS absent to mild: VSS 0-2 moderate to severe: VSS 3-4 | CT | sICH: hemorrhagic transformation that caused clinical deterioration of 4 points or more on the NIHSS within 36 hours | EVT |
| Drelon | 2020 | France | retrospective | 944 | NA | 75 | AIS | Not | Not | 8 | MRI(FLAIR/T2/ time-of-flight magnetic resonance angiographic sequences) | Fazekas | CT | rICH: Heidelberg Bleeding Classification | IVT |
| Sanchez | 2017 | Spain | retrospective | 384 | NA | NA | AIS | Not | Not | 10 | MRI(FLAIR/T2/gradient-recalled echo sequences or sensitive to magnetic susceptibility) | Fazekas score of 2 (periventricular smooth halo or deep beginning confluent foci) or 3 (periventricular irregular lesions extending into deep white matter or deep confluent lesions) | MRI | rPH: ECASS I criteria | IVT |
| Singer | 2008 | Germany | retrospective | 536 | NA | 68(57-76) | AIS | Not | anterior circulation stroke | 7 | MRI(DWI) | Fazekas | CT/MRI | sICH: ECASS I criteria | IVT |
| Eryildiz | 2020 | Turkey | retrospective | 302 | 57.8 | 67 | AIS | Not | anterior circulation stroke | 15 | CT | VSS severe: VSS>4 | CT | sICH: ECASS II criteria | IVT |
| Mutzenbach | 2020 | USA | retrospective | 209 | 46.9 | 75.0(63.0-81.0) | AIS | LVO | anterior circulation stroke | 17(14-21) | CT/MRI(T2/FLAIR) | ARWMC  top 25 percentiles of LASs were categorized as severe LA and others as absent/mild LA | CT/MRI | sICH: Worsening of National Institute of Health Stroke Scale (NIHSS) score no less than 4 points and the presence of ICH | EVT |
| Aries | 2010 | Belgium | retrospective | 400 | 53 | 68(14) | AIS | Not | Not | 13(8-17) | CT | tVSS moderate: tVSS 1-4 severe: tVSS > 4 | CT | sICH: SITS-MOST definition | IVT |
| Mistry | 2020 | USA | retrospective | 389 | 50 | 67.9 | AIS | LVO | anterior circulation stroke | 15 | CT | VSS non to minimal: VSS 0-2 moderate to severe: VSS 3-4 | CT | sICH: 4 points or more increase in the NIHSS score from baseline to 24 hours | EVT |
| Curtze(2) | 2015 | Finland | retrospective | 2485 | 56.7 | 69 | AIS | Not | Not | 8 | CT | VSS, Blennow, Wahlund severe: VSS 3-4 | CT | sICH: ECASS II defination | IVT |
| Brainer | 2022 | Brazil | retrospective | 988 | 60.4 | 67.95 | AIS | Not | Not | 13.9 | CT/MRI | NA | CT/MRI | HT: ECASS II criteria | None |
| Wang | 2022 | China | retrospective | 190 | 74.2 | 64.84 | AIS | Not | Not | NA | MRI | Fazekas | CT/MRI | HT: ECASS II criteria | None |
| Note: IVT, intravenous thrombolysis; EVT, endovascular therapy; AC, anticoagulants; AP, antiplatelets; None, IVT or EVT were not used | | | | | | | | | | | | | | | |

1. **Supplemental Table 2.** Quality assessment

In this study, the quality of included researches were assessed based on Newcastle-Ottawa Scale (NOS), a tool for assessing risk of bias in observational studies. We chose NOS for cohort studies and the criteria for every scale item was as follow:

Note: A study can be awarded a maximum of one star for each numbered item within the Selection and Outcome categories. A maximum of two stars can be given for Comparability

**Selection**

1) Representativeness of the exposed cohort

a) truly representative of the average stroke patients with WML at baseline in the community

b) somewhat representative of the average stroke patients with WML at baseline in the community

c) selected group of users eg nurses, volunteers

d) no description of the derivation of the cohort

A point was award if the study included stroke patients with WML at baseline and recruited either from the general population or consecutively from hospital-based setting.

2) Selection of the non exposed cohort

a) drawn from the same community as the exposed cohort

b) drawn from a different source

c) no description of the derivation of the non exposed cohort

A point was award if the stroke patients without WML were selected from the same source with patients with WML.

3) Ascertainment of exposure

a) secure record (eg surgical records)

b) structured interview

c) written self report

d) no description

A point was award if the WML was assessed via quantitative scale, eg. Fazekas Scale, Van Swieten Scale, etc.

4) Demonstration that outcome of interest was not present at start of study

a) yes

b) no

A point was awarded if there were no outcome measures determined to observe at the start of the study.

**Comparability**

1) Comparability of cohorts on the basis of the design or analysis

a) study controls for age

b) study controls for any additional factor

A point was awarded if the study had adjusted the presented effect estimates for age. And one additional point was awarded if the effect estimates were adjusted for additional factors.

**Outcome**

1) Assessment of outcome

a) independent blind assessment

b) record linkage

c) self report

d) no description

A point was awarded if the outcome (any type of HT) was assessed via established criteria, eg. ECASS criteria or had record linkage.

2) Was follow-up long enough for outcomes to occur

a) yes

b) no

A point was awarded if any type of HT was assessed after reperfusion therapy and within 14 days after treatment.

3) Adequacy of follow up of cohorts

a) complete follow up - all subjects accounted for

b) subjects lost to follow up unlikely to introduce bias - small number lost - > 10 %

c) follow up rate < 90% and no description of those lost

d) no statement

A point was awarded if completion of follow-up was clearly reported and attrition rates were < 10%.

| **Study**  **(Author-year)** | **Selection items** | | | | **Comparability items** | | **Outcome items** | | | **Total score** |
| --- | --- | --- | --- | --- | --- | --- | --- | --- | --- | --- |
|  | Representativeness of the exposed cohort | Selection of the non exposed cohort | Ascertainment of exposure | Outcome not present at start of study | Age | Additional adjustments | Assessment of outcome | Follow-up length | Adequacy of follow up of cohorts |  |
| Costello-2012 | 1 | 1 | 1 | 1 | 0 | 1 | 1 | 1 | 0 | 7 |
| Zheng-2012 | 1 | 1 | 1 | 0 | 0 | 0 | 1 | 1 | 0 | 5 |
| Wardlaw-2015 | 0 | 1 | 1 | 1 | 1 | 1 | 1 | 1 | 1 | 8 |
| Wei(1)-2017 | 1 | 1 | 1 | 1 | 1 | 1 | 1 | 1 | 1 | 9 |
| Luijten-2021 | 1 | 1 | 1 | 0 | 1 | 1 | 0 | 1 | 0 | 6 |
| Delcourt-2020 | 1 | 1 | 1 | 1 | 1 | 1 | 1 | 1 | 1 | 9 |
| Curtze(1)-2016 | 1 | 1 | 1 | 1 | 1 | 1 | 1 | 1 | 1 | 9 |
| Willer-2015 | 1 | 1 | 1 | 1 | 1 | 1 | 0 | 1 | 0 | 7 |
| Liu(1)-2018 | 1 | 1 | 1 | 1 | 1 | 1 | 1 | 1 | 0 | 8 |
| Cho-2012 | 1 | 1 | 1 | 1 | 1 | 1 | 1 | 1 | 0 | 8 |
| Kufner-2013 | 1 | 1 | 1 | 1 | 0 | 0 | 1 | 1 | 0 | 6 |
| Anna-2021 | 1 | 1 | 1 | 1 | 0 | 1 | 1 | 1 | 1 | 8 |
| Demchuk-2008 | 1 | 1 | 1 | 1 | 1 | 1 | 1 | 1 | 0 | 8 |
| Benson-2021 | 1 | 1 | 1 | 1 | 1 | 1 | 1 | 0 | 0 | 7 |
| Palumbo-2007 | 1 | 1 | 1 | 1 | 1 | 1 | 0 | 1 | 0 | 7 |
| Yang-2018 | 1 | 1 | 1 | 1 | 1 | 1 | 1 | 1 | 1 | 9 |
| Neumann-haefelin-2006 | 1 | 1 | 1 | 1 | 1 | 0 | 1 | 0 | 0 | 6 |
| Liu(2)-2019 | 1 | 1 | 1 | 1 | 1 | 1 | 1 | 1 | 0 | 8 |
| Choi-2011 | 1 | 1 | 1 | 1 | 1 | 1 | 1 | 1 | 0 | 8 |
| Shi-2012 | 1 | 1 | 1 | 1 | 0 | 0 | 1 | 1 | 0 | 6 |
| Guo-2019 | 1 | 1 | 1 | 1 | 1 | 1 | 1 | 1 | 0 | 8 |
| Yanez-2006 | 0 | 0 | 1 | 1 | 0 | 1 | 1 | 0 | 0 | 4 |
| Kawano-2012 | 1 | 1 | 1 | 1 | 0 | 0 | 1 | 1 | 0 | 6 |
| Nawar-2019 | 1 | 1 | 1 | 1 | 1 | 1 | 1 | 0 | 0 | 7 |
| Wei(2)-2019 | 1 | 1 | 1 | 1 | 1 | 1 | 1 | 1 | 0 | 8 |
| Albo-2021 | 1 | 1 | 1 | 1 | 1 | 1 | 1 | 1 | 0 | 8 |
| Drelon-2020 | 1 | 1 | 1 | 1 | 1 | 1 | 1 | 1 | 0 | 8 |
| Sanchez-2017 | 1 | 1 | 1 | 1 | 1 | 1 | 1 | 1 | 0 | 8 |
| Singer-2008 | 1 | 1 | 1 | 1 | 1 | 1 | 1 | 1 | 1 | 9 |
| Eryildiz-2020 | 1 | 1 | 1 | 1 | 0 | 0 | 1 | 0 | 0 | 5 |
| Mutzenbach-2020 | 1 | 1 | 1 | 1 | 1 | 1 | 0 | 1 | 0 | 6 |
| Aries-2010 | 1 | 1 | 1 | 1 | 1 | 1 | 1 | 0 | 0 | 7 |
| Mistry-2020 | 1 | 1 | 1 | 1 | 1 | 1 | 0 | 1 | 1 | 8 |
| Curtze(2)-2015 | 1 | 1 | 1 | 1 | 1 | 1 | 1 | 1 | 0 | 8 |
| Brainer-2022 | 1 | 1 | 1 | 1 | 1 | 1 | 1 | 1 | 0 | 8 |
| Wang-2022 | 1 | 1 | 1 | 1 | 0 | 0 | 1 | 1 | 0 | 6 |

1. **Supplemental Figure 1.** Subgroup analysis of presence of WML and risk of HT


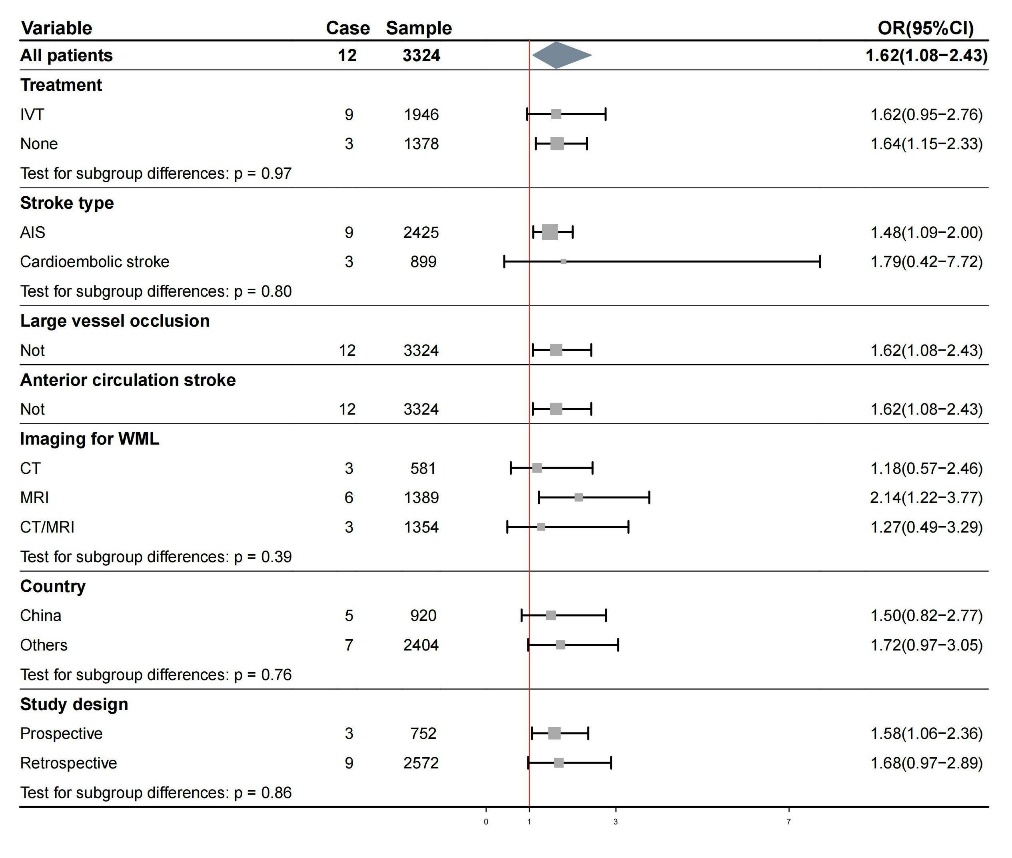


1. **Supplemental Figure 2.** Subgroup analysis of moderate-to-severe WML and risk of HT


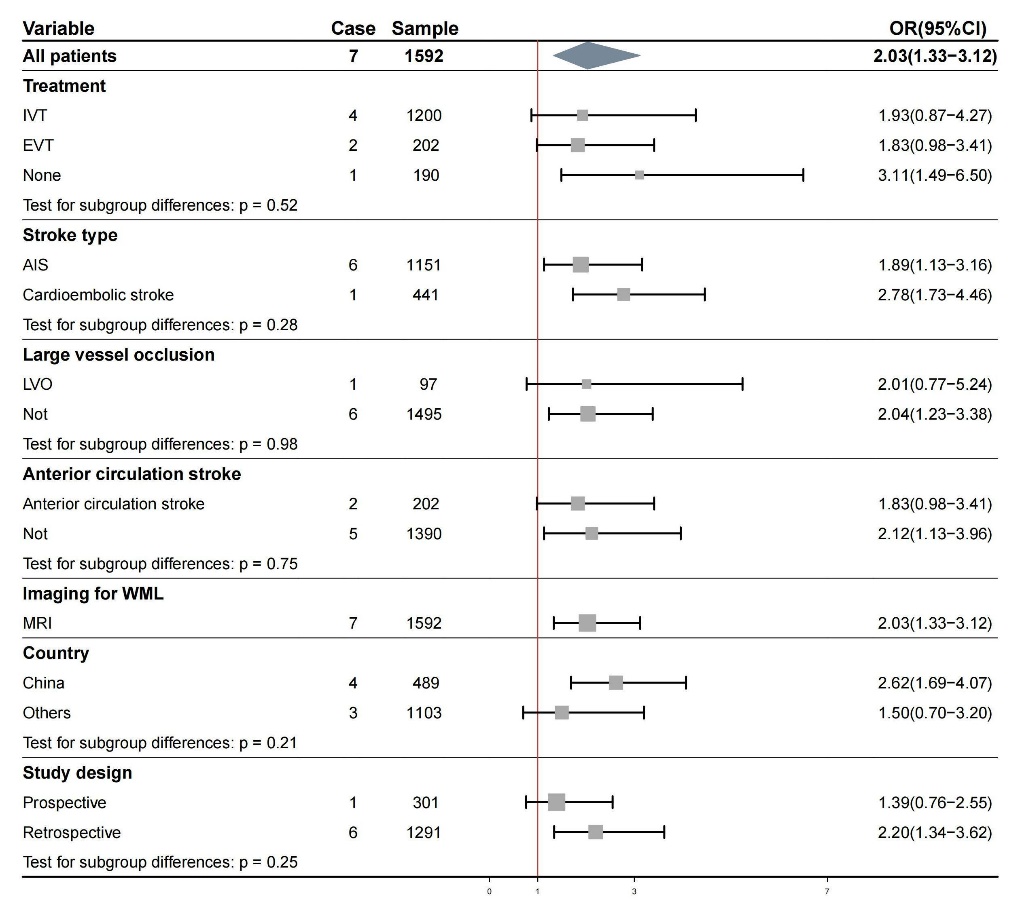


1. **Supplemental Figure 3.** Subgroup analysis of severe WML and risk of HT


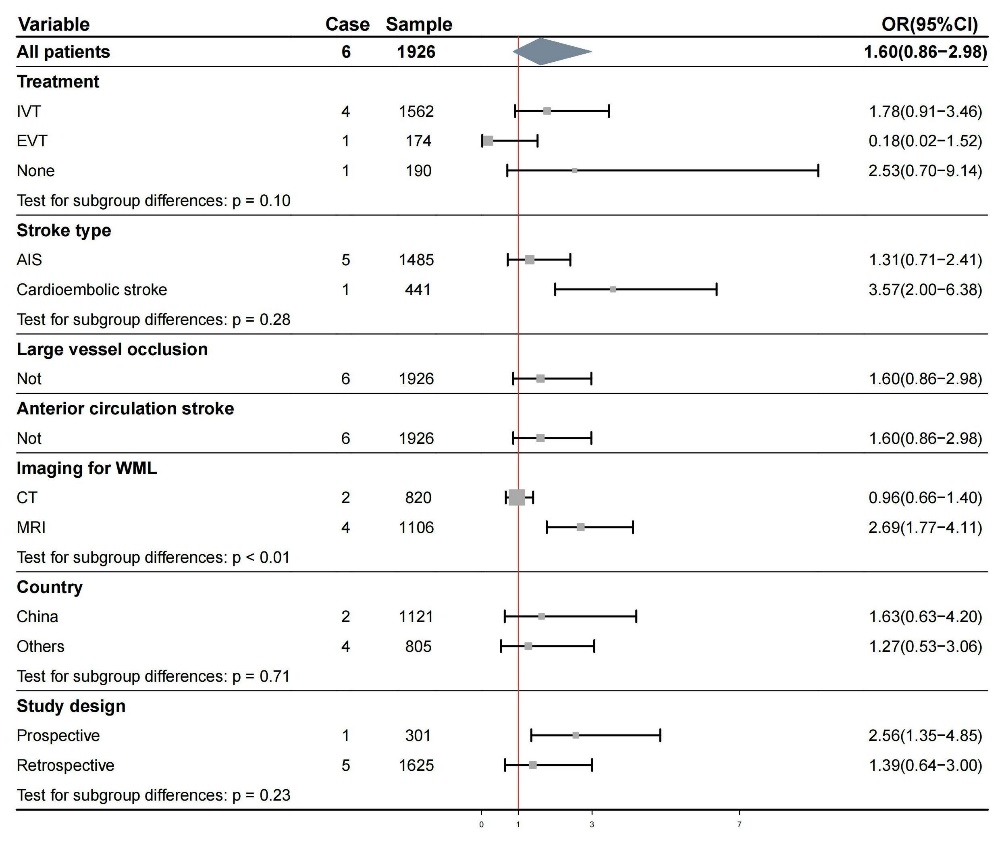


1. **Supplemental Figure 4.** Subgroup analysis of presence of WML and risk of sICH


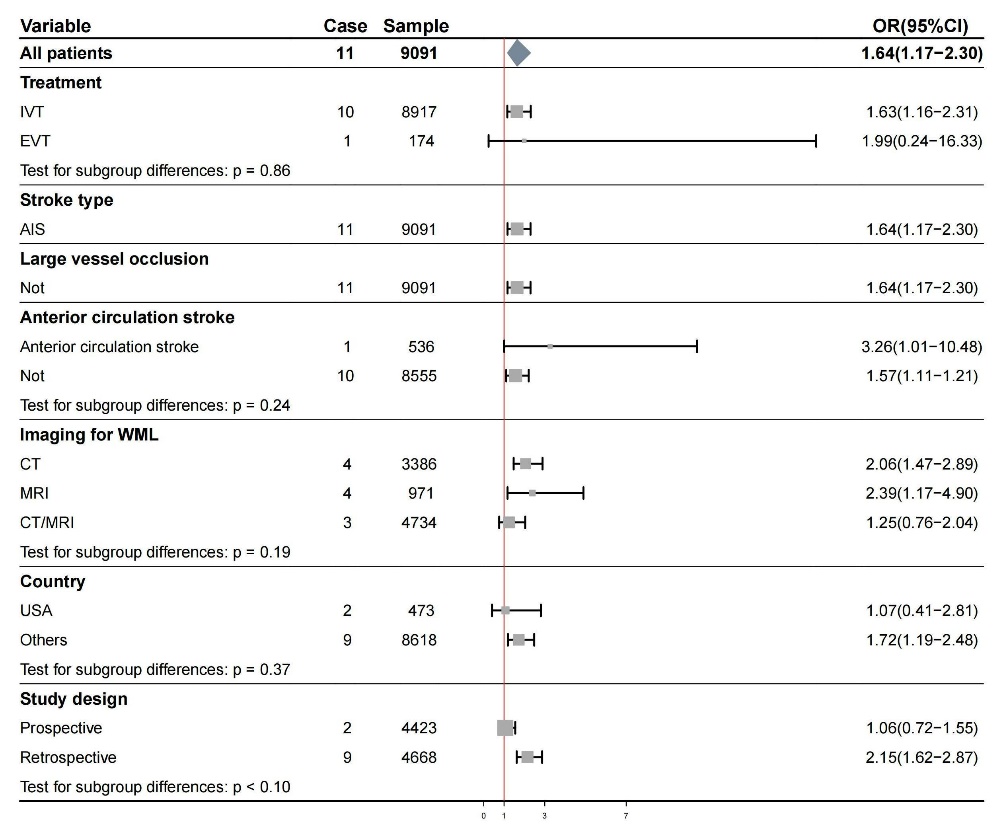


1. **Supplemental Figure 5.** Subgroup analysis of moderate-to-severe WML and risk of sICH


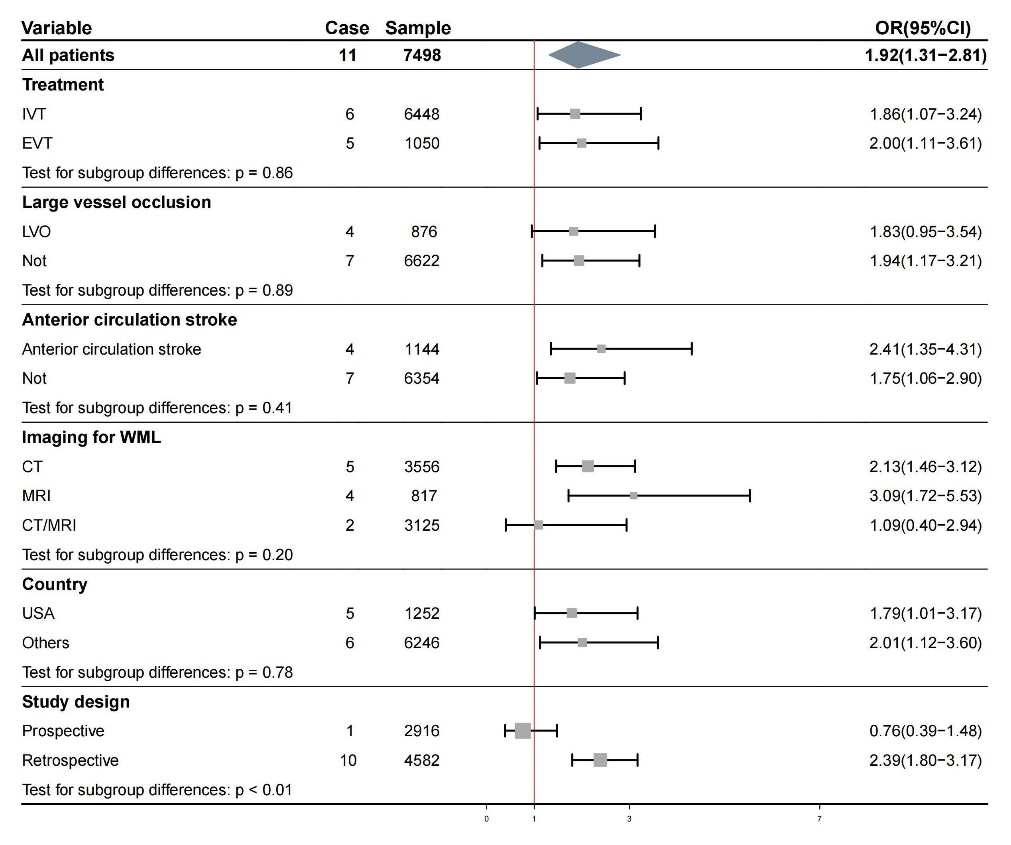


1. **Supplemental Figure 6.** Subgroup analysis of severe WML and risk of sICH


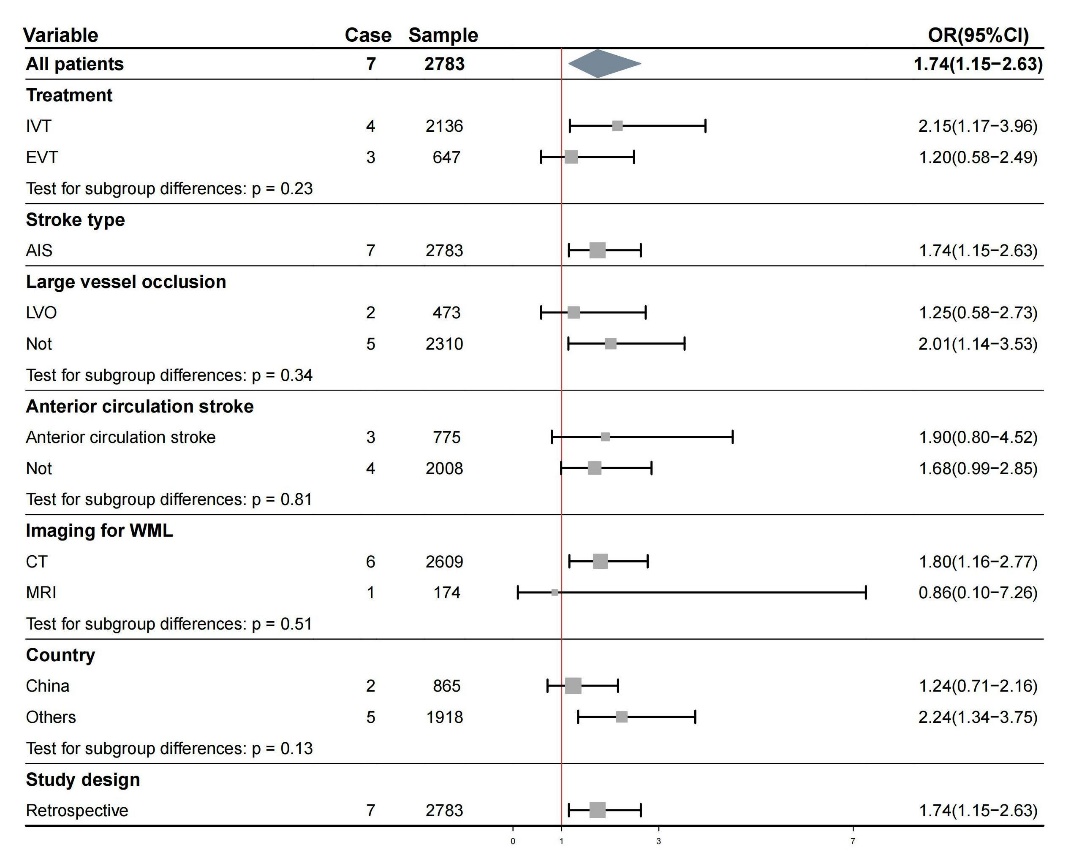


1. **Supplemental Figure 7.** Funnel plot of included studies


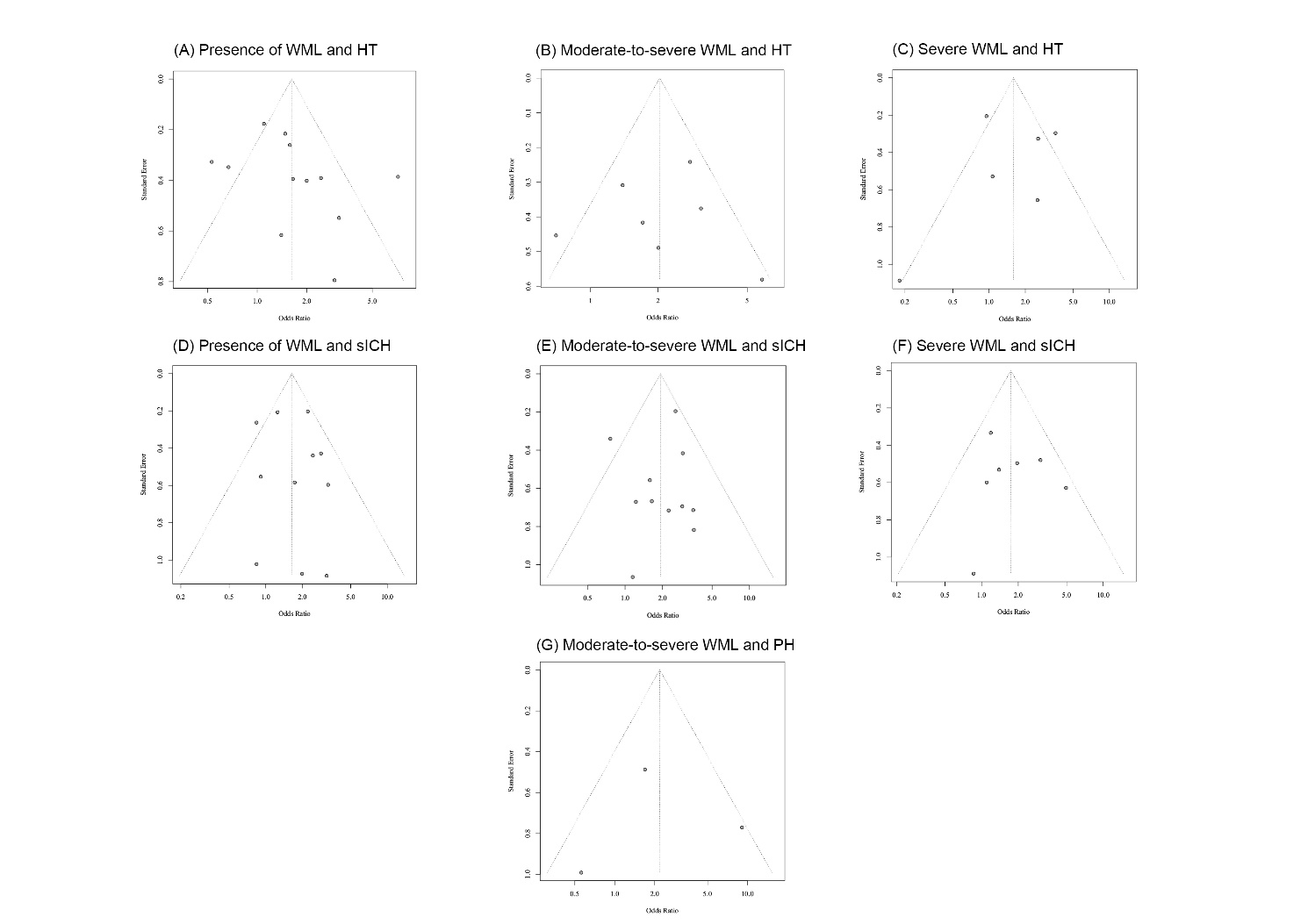


1. **Supplemental Figure 8.** Egger’s test


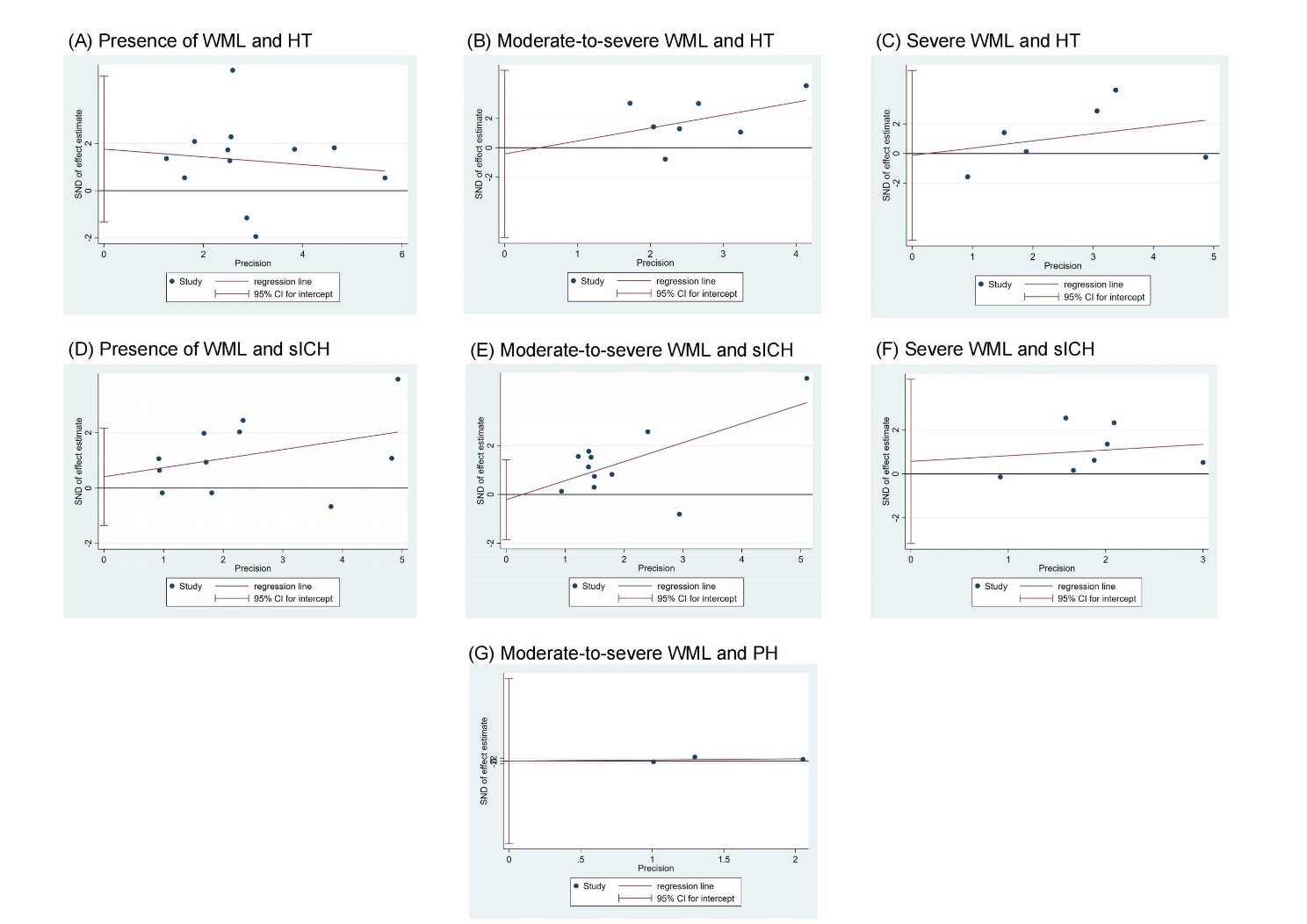


1. **Supplemental Figure 9.** Sensitivity analysis of meta-analysis (influence of a single study)


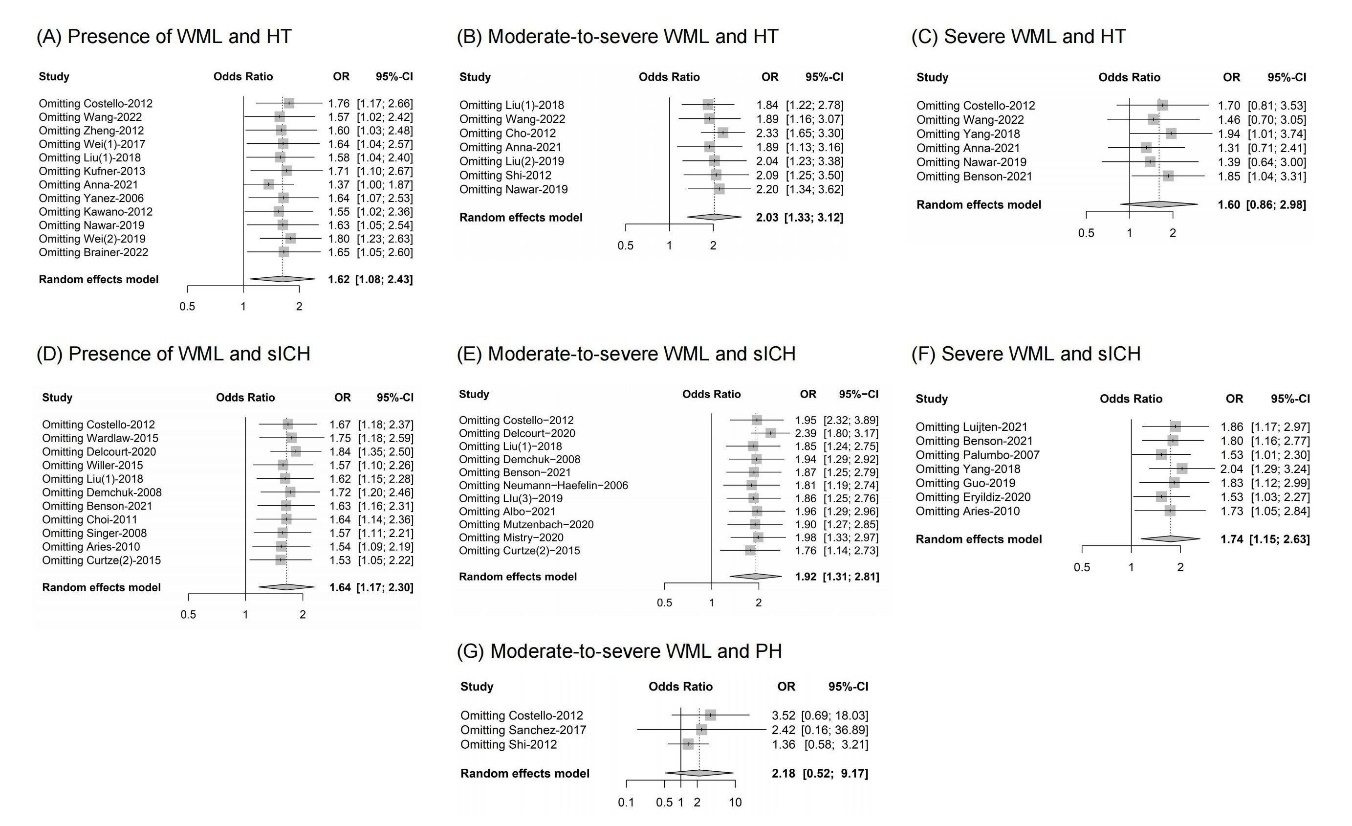


1. **PRISMA 2020 checklist**

| **Section and Topic** | **Item #** | **Checklist item** | **Location where item is reported** |
| --- | --- | --- | --- |
| **TITLE** | | |  |
| Title | 1 | Identify the report as a systematic review. | P1 |
| **ABSTRACT** | | |  |
| Abstract | 2 | See the PRISMA 2020 for Abstracts checklist. | P2 |
| **INTRODUCTION** | | |  |
| Rationale | 3 | Describe the rationale for the review in the context of existing knowledge. | P4/Line2-17 |
| Objectives | 4 | Provide an explicit statement of the objective(s) or question(s) the review addresses. | P5/Line33-36 |
| **METHODS** | | |  |
| Eligibility criteria | 5 | Specify the inclusion and exclusion criteria for the review and how studies were grouped for the syntheses. | P6/Line51-58 |
| Information sources | 6 | Specify all databases, registers, websites, organisations, reference lists and other sources searched or consulted to identify studies. Specify the date when each source was last searched or consulted. | P5-6/Line44-49 |
| Search strategy | 7 | Present the full search strategies for all databases, registers and websites, including any filters and limits used. | P5-6/Line44-49 |
| Selection process | 8 | Specify the methods used to decide whether a study met the inclusion criteria of the review, including how many reviewers screened each record and each report retrieved, whether they worked independently, and if applicable, details of automation tools used in the process. | P6-7/Line65-77 |
| Data collection process | 9 | Specify the methods used to collect data from reports, including how many reviewers collected data from each report, whether they worked independently, any processes for obtaining or confirming data from study investigators, and if applicable, details of automation tools used in the process. | P6-7/Line65-77 |
| Data items | 10a | List and define all outcomes for which data were sought. Specify whether all results that were compatible with each outcome domain in each study were sought (e.g. for all measures, time points, analyses), and if not, the methods used to decide which results to collect. | P6-7/Line65-77 |
|  | 10b | List and define all other variables for which data were sought (e.g. participant and intervention characteristics, funding sources). Describe any assumptions made about any missing or unclear information. | P6-7/Line65-77 |
| Study risk of bias assessment | 11 | Specify the methods used to assess risk of bias in the included studies, including details of the tool(s) used, how many reviewers assessed each study and whether they worked independently, and if applicable, details of automation tools used in the process. | P7/Line91 |
| Effect measures | 12 | Specify for each outcome the effect measure(s) (e.g. risk ratio, mean difference) used in the synthesis or presentation of results. | P7/Line82-83 |
| Synthesis methods | 13a | Describe the processes used to decide which studies were eligible for each synthesis (e.g. tabulating the study intervention characteristics and comparing against the planned groups for each synthesis (item #5)). | P7/Line79-92 |
|  | 13b | Describe any methods required to prepare the data for presentation or synthesis, such as handling of missing summary statistics, or data conversions. | P7/Line79-92 |
|  | 13c | Describe any methods used to tabulate or visually display results of individual studies and syntheses. | P7/Line79-92 |
|  | 13d | Describe any methods used to synthesize results and provide a rationale for the choice(s). If meta-analysis was performed, describe the model(s), method(s) to identify the presence and extent of statistical heterogeneity, and software package(s) used. | P7/Line79-92 |
|  | 13e | Describe any methods used to explore possible causes of heterogeneity among study results (e.g. subgroup analysis, meta-regression). | P7/Line79-92 |
|  | 13f | Describe any sensitivity analyses conducted to assess robustness of the synthesized results. | P7/Line79-92 |
| Reporting bias assessment | 14 | Describe any methods used to assess risk of bias due to missing results in a synthesis (arising from reporting biases). | P7/Line79-92 |
| Certainty assessment | 15 | Describe any methods used to assess certainty (or confidence) in the body of evidence for an outcome. | NA |
| **RESULTS** | | |  |
| Study selection | 16a | Describe the results of the search and selection process, from the number of records identified in the search to the number of studies included in the review, ideally using a flow diagram. | P8-9/Line103-118 |
|  | 16b | Cite studies that might appear to meet the inclusion criteria, but which were excluded, and explain why they were excluded. | P8-9/Line103-118 |
| Study characteristics | 17 | Cite each included study and present its characteristics. | P8-9/Line103-118 |
| Risk of bias in studies | 18 | Present assessments of risk of bias for each included study. | P11/Line177-181 |
| Results of individual studies | 19 | For all outcomes, present, for each study: (a) summary statistics for each group (where appropriate) and (b) an effect estimate and its precision (e.g. confidence/credible interval), ideally using structured tables or plots. | P9/Line119-129 |
| Results of syntheses | 20a | For each synthesis, briefly summarise the characteristics and risk of bias among contributing studies. | P9-P11/Line131-175 |
|  | 20b | Present results of all statistical syntheses conducted. If meta-analysis was done, present for each the summary estimate and its precision (e.g. confidence/credible interval) and measures of statistical heterogeneity. If comparing groups, describe the direction of the effect. | P9-P11/Line131-175 |
|  | 20c | Present results of all investigations of possible causes of heterogeneity among study results. | P9-P11/Line131-175 |
|  | 20d | Present results of all sensitivity analyses conducted to assess the robustness of the synthesized results. | P11/Line177-181 |
| Reporting biases | 21 | Present assessments of risk of bias due to missing results (arising from reporting biases) for each synthesis assessed. | P11/Line177-181 |
| Certainty of evidence | 22 | Present assessments of certainty (or confidence) in the body of evidence for each outcome assessed. | NA |
| **DISCUSSION** | | |  |
| Discussion | 23a | Provide a general interpretation of the results in the context of other evidence. | P12/Line192-196 |
|  | 23b | Discuss any limitations of the evidence included in the review. | P18/Line331-336 |
|  | 23c | Discuss any limitations of the review processes used. | P18/Line331-336 |
|  | 23d | Discuss implications of the results for practice, policy, and future research. | P18-19/Line337-348 |
| **OTHER INFORMATION** | | |  |
| Registration and protocol | 24a | Provide registration information for the review, including register name and registration number, or state that the review was not registered. | P5/Line40-43 |
|  | 24b | Indicate where the review protocol can be accessed, or state that a protocol was not prepared. | P5/Line40-43 |
|  | 24c | Describe and explain any amendments to information provided at registration or in the protocol. | P5/Line40-43 |
| Support | 25 | Describe sources of financial or non-financial support for the review, and the role of the funders or sponsors in the review. | P19/Line350-352 |
| Competing interests | 26 | Declare any competing interests of review authors. | P19/Line353-354 |
| Availability of data, code and other materials | 27 | Report which of the following are publicly available and where they can be found: template data collection forms; data extracted from included studies; data used for all analyses; analytic code; any other materials used in the review. | P19/Line355-357 |

*From:*  Page MJ, McKenzie JE, Bossuyt PM, Boutron I, Hoffmann TC, Mulrow CD, et al. The PRISMA 2020 statement: an updated guideline for reporting systematic reviews. BMJ 2021;372:n71. doi: 10.1136/bmj.n71
